# Team-Based Learning & Point of Care Ultrasound (POCUS) to Augment a Preclinical Cardiovascular Physiology Course

**DOI:** 10.24908/pocus.v9i2.17241

**Published:** 2024-11-15

**Authors:** Mark Danila, Cynthia Zheng, Ryan J Salvatore, Rachel Cary, Sara Youssef, Grace Pinhal-Eenfield, Catherine Chen

**Affiliations:** 1 Department of Anesthesiology and Critical Care, Hospital of the University of Pennsylvania Philadelphia, PA USA; 2 Department of Medicine, The Warren Alpert Medical School of Brown University Providence, RI USA; 3 Rutgers Robert Wood Johnson Medical School Piscataway, NJ USA; 4 Department of Neuroscience and Cell Biology, Rutgers Robert Wood Johnson Medical School Piscataway, NJ USA; 5 Department of Medicine, Rutgers Robert Wood Johnson Medical School New Brunswick, NJ USA

**Keywords:** Education, Undergraduate Clerkship, Preclinical, Point of Care Echocardiography, Flipped Classroom

## Abstract

**Introduction:** There has been increasing interest in point of care ultrasound (POCUS) as a learning tool in preclinical medical anatomy and physiology courses. Few interventions have used team-based learning (TBL) to teach cardiac POCUS. This study investigates a novel TBL exercise designed to integrate cardiac anatomy, physiology, and cardiac POCUS education within a first-year cardiovascular (CV) course called Team-Based Learning – Ultrasound (TBL-US). **Methods**: The TBL-US exercise consisted of four phases: preparation, individual and team readiness assurance, image acquisition and application, and knowledge assessment. Six second-year students were trained to facilitate the session under physician supervision. Pre- and post-session knowledge assessments were administered to determine knowledge acquisition. Pre- and post-session surveys were administered to assess attitudes, beliefs, and confidence surrounding cardiac POCUS. Final exam scores were compared between participants and non-participants of TBL-US and stratified into high- and low-performing subgroups to account for pre-TBL baseline differences in ability between the groups. **Results**: A total of 54 first-year medical students completed TBL-US. Students showed significant improvement on the post-knowledge assessment compared to the pre-knowledge assessment (70.5% vs. 54.9% [p< 0.001]) and scored significantly higher on the final CV exam compared to non-participants (low-performing group: 85.92% vs. 81.02% [p=0.039], high-performing group: 89.22% vs. 85.95% [p=0.038]). Between 43.3-72.7% of students reported that TBL-US increased their understanding of CV anatomy, physiology, and cardiac POCUS. **Discussion**: Students found TBL-US to be a valuable teaching modality and improved student knowledge of CV anatomy, physiology, and cardiac POCUS. TBL-US effectively augments the learning of cardiac anatomy and physiology during the preclinical undergraduate medical curriculum.

## Background

Point of care ultrasound (POCUS) is becoming the standard-of-care in many specialties as a clinical decision-making tool due to its accessibility, low cost, and noninvasiveness 1. There is an increased demand for POCUS education in residency training 2. Due to the density of residency curricula, residents may not have adequate time to train properly in POCUS 1, 2, 3, 4, 5, 6. In response, there has been growing incorporation of POCUS education into undergraduate medical education, specifically in the preclinical years 2, 3, 4, 6, 7, 8, 9, 10, 11, 12, 13, 14.

POCUS can be integrated effectively into undergraduate medical education and tailored to specific organ systems 13, 15, 16, 17. For example, the inclusion of POCUS into preclinical cardiovascular (CV) physiology courses has the potential to enhance the learning of cardiac anatomy and physiology 4, 10, 11, 12, 13, 17, 18. Unsurprisingly, many medical students learning cardiac POCUS and abdominal POCUS have agreed that ultrasound education improved their understanding and retention of basic science concepts, likely because of gap bridging between conceptual knowledge and practical application 1, 7, 10, 11, 12, 13, 15, 18, 19.

Despite a movement toward POCUS integration into preclinical undergraduate medical education, a standardized teaching approach is lacking 2, 4, 6, 13. Several medical schools have endorsed a flipped classroom model for POCUS education due to its efficiency 1, 3, 4, 5, 6, 8, 10, 11, 12. Team-based learning (TBL) utilizes this flipped classroom approach to encourage teamwork, communication, and collaboration through active learning. TBL has proven to be an effective educational method that fosters collaborative learning through peer-to-peer discussions supported by facilitator guidance 20.

During the initial preparation phase, students complete an assignment before class to ensure adequate preparation for active participation in the next phase. In thereadiness assurance phase, learners engage in activities to test their understanding individually through an Independent Readiness Assurance Test (IRAT), followed by a team assessment during the Group Readiness Assurance Test (GRAT). The GRAT promotes interactive peer-to-peer discussions of key concepts. Facilitators provide guidance only after teams have attempted to answer questions among learners. For the application phase, teams apply their knowledge and understanding from the previous phases to solve cases with subsequent reporting in a classroom discussion. This class discussion provides immediate feedback on more challenging issues 20, 21.

Overall, students reported that TBL enhances their learning and improves their confidence when compared to traditional education methodologies 19, 22, 23, 24, 25, 26. In our study, TBL was used to teach POCUS, utilizing its phases and collaborative approach to enhance learning outcomes in practical skills training. Few studies have assessed the efficacy of POCUS education with TBL. Previous literature demonstrates that TBL can be superior to traditional education methodologies in teaching musculoskeletal POCUS skills and non-inferior to traditional education methodologies in teaching cardiac echocardiography skills to undergraduate medical students 27, 28. No intervention is reported in the literature that has specifically evaluated POCUS with TBL to augment a preclinical CV physiology course. Thus, a novel teaching session termed Team-Based Learning – Ultrasound (TBL-US) was developed and delivered to first-year medical students during their CV physiology course to connect CV anatomy and physiology using POCUS.

## Methods

### Study Setting

This was a pre-post study that sought to determine whether an educational workshop on cardiac POCUS could improve knowledge of CV anatomy and physiology among first-year medical students. 

Robert Wood Johnson Medical School is affiliated with an urban academic medical center and provides a Medical Doctorate (MD) upon graduation. The workshop was run in the fall of 2019. At the time of the study, no formal POCUS training was available to preclinical medical students.

**Figure 1:**
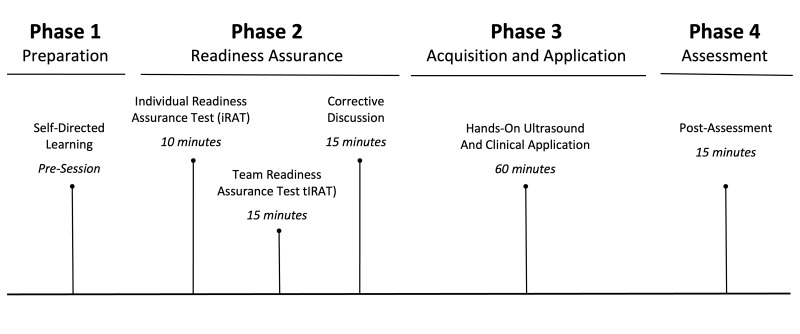
TBL-US Timeline. Overview of the Team Based Learning-Ultrasound (TBL-US) phases.

All first-year students who were actively enrolled in the first-year CV physiology course were eligible to sign up for the TBL-US session during their CV physiology course. For recruitment, M.D. and C.Z. presented the TBL-US session earlier in the CV physiology course to students and sent emails with sign-up instructions. The entire TBL-US session lasted two hours and included four phases: preparation, individual and team readiness assurance, image acquisition and application, and knowledge assessment. Figure 1 depicts the overall timeline. This intervention was approved by the Institutional Review Board of the Rutgers Biomedical and Health Sciences (Pro2019002087).

### Study Design

In Phase 1, students completed a virtual self-directed learning module that introduced cardiac anatomy, physiology, and cardiac POCUS one week before the in-person session. The virtual self-directed learning module was created by the authors using a variety of resources, including video clips from free open access medical education (FOAMed) sources such as 5 Minute SONO and POCUS images available on Creative Commons (Supplemental). Phases 2 through 4 comprised the in-person session.

In Phase 2, students individually completed an IRAT that assessed POCUS concepts and cardiac physiology. The IRAT served as the pre-knowledge assessment test. All questions were multiple choice and written by M.D. and C.Z. Content validity was assessed by the CV physiology course director (G.P.E.) and a physician with POCUS and medical education expertise (C.C.). Students also completed a pre-session survey that assessed attitudes, beliefs, and confidence regarding cardiac POCUS. Following completion of the IRAT, students were randomly sorted into groups of five and completed the same seven-question assessment, now called the GRAT. Students were then provided with a packet containing the answers with their explanations. 

In Phase 3, students were provided with one hour of hands-on cardiac POCUS instruction and application with volunteer models, who were undergraduate students referred from the university's health professions office. Near-peer facilitators demonstrated the four cardiac views: parasternal long and short axis, apical four-chamber, and subxiphoid, and were given a reference guide and sheet that included anatomy images and clinical correlations. Students individually practiced obtaining all views at least twice. In Phase 4, students completed a post-session knowledge assessment, which tested the same concepts as the IRAT, and a post-session survey. Two faculty members were available throughout the session. Full materials are available in the appendices. Non-participants were not provided with any material or resources from any of the TBL-US phases.

### Near-Peer Facilitators

Second-year medical students who completed their first-year preclinical CV physiology course served as near-peer facilitators. Before facilitating, they completed the same Self-Directed Learning module as participating students and attended three POCUS training sessions. At the end of the first session, they completed a practical exam during which they demonstrated the four standard cardiac POCUS views on each volunteer twice. The other sessions served as opportunities for additional practice.

### Cardiovascular Physiology Final Exam

The TBL-US session occurred after all formal CV physiology lectures were completed and one week prior to the multiple-choice final exam. The CV physiology final exam scores were used as a surrogate measure for retention of knowledge. The course coordinator collected CV final exam scores. Fifty-four students who had not participated in the TBL-US workshop were randomly selected as the “non-participant” group. To control for differences in pre-intervention scholastic performance and minimize participation bias, both groups were further stratified into “high-performer” and “low-performer” subgroups based on whether they scored above or at/below the class average on the “Structure and Function” anatomy exam one month prior. No questions from the IRAT were used in the final exam. 

**Figure 2:**
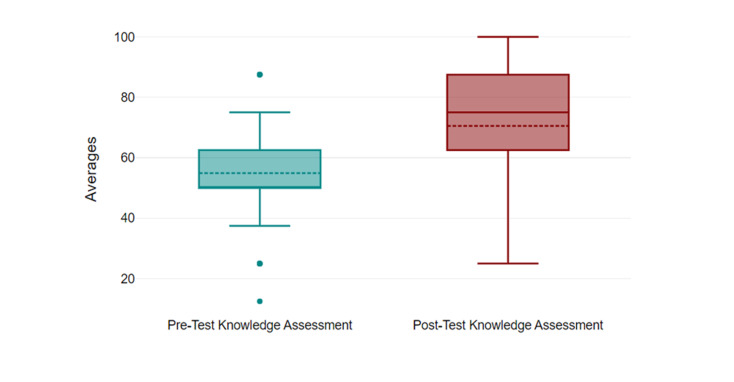
Knowledge Assessments Results. Overall, post-knowledge mean assessment scores (70.5%) were significantly higher compared to pre-knowledge assessment scores (54.9%) (p<0.001). Post-knowledge assessment scores were also higher when subdivided by key concepts tested for combinedPOCUS and cardiac physiology (p<0.001), cardiac physiology (p<0.001), and POCUS (p=0.029).

**Figure 3:**
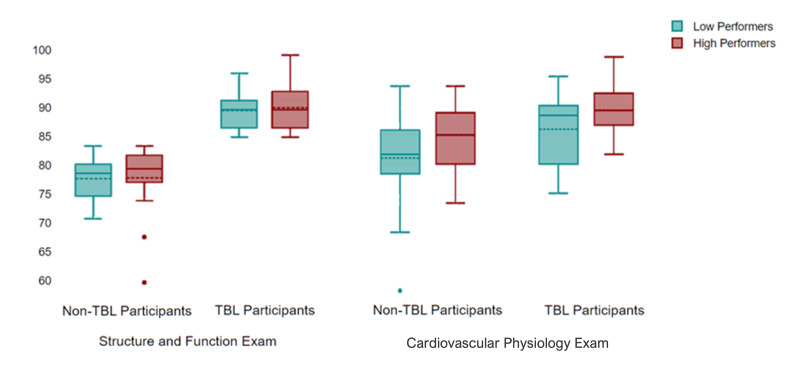
Cardiovascular Physiology Exam Scores. The box-plots of the mean structure and function exam score were used to stratify students into high- and low- performing groups. Students who scored above the mean were considered “high-performers” while students who scored at or below the mean were considered “low-performers.” TBL-US participants scored significantly higher on the final exam than non-TBL-US participants in both performance stratifications (high performers p = 0.038; low-performers p = 0.039).

### Data Collection & Analysis 

For the pre- and post-session knowledge assessments, data was collected anonymously through Qualtrics survey tool via electronic mobile devices and was therefore unpaired. Since the Kolmogorov-Smirnov (KS) test of normality indicated a non-normal distribution, the Mann-Whitney U test was used to determine significance between the medians due to the nature of the data collection.

For the CV exam results, the KS test showed mixed results of normality and Q-Q plot visualization was used to determine normal distributions of the participant and nonparticipant branches for the Structure and Function and the CV Final Exam scores. As a result, student t-tests were conducted to determine statistical significance. Adjusted and unadjusted multivariable linear regression was performed to limit the effect of a potential confounder – previous performance on the Structure and Function exam. 

For the pre- and post- surveys which included a mix of 5-point Likert scales and descriptive open-ended questions, the results were analyzed via a paired Wilcoxon Signed-Rank nonparametric test. Due to the number of tests performed – 11 tests – a Bonferroni correction was used to reduce type I error. 

## Results

There were 172 eligible first year medical students, of which 49.1% were female and 50.9% were male. Students were only assigned to one ethnicity based on coding, including 39.4% self-identified as Asian, 34.5% as White, 9.7% as Hispanic, 8.5% as Black/African American, and 7.9% as other/undeclared. A total of 54 students completed the TBL-US session. 

### Knowledge Assessment Results (IRAT)

All 54 participants completed the pre- and post-knowledge readiness assessments. The overall post-assessment median score of 75% (Interquartile Range 62.5%-87.5%) was significantly higher than the median pre-assessment score of 50% (Interquartile Range 50%-62.5%) (p< 0.001). Post-knowledge assessment scores were also higher than pre-assessment knowledge assessment scores when subdivided by key concepts tested: POCUS (post=84.9%; pre=72.59%; p=0.029), cardiac physiology (post=74.84%; pre=49.67%; p<0.001), and combined POCUS and cardiac physiology (post=71.79%; pre=39.21%; p<0.001) (Figure 2). 

## Final Examination Results

Figure 3 depicts the CV physiology final exam scores. Fifty-two students had their exam scores analyzed (2 students deferred their CV exams). The structure and function (anatomy) final exam, taken prior to the TBL and CV physiology final exam, had a class mean of 82.17%. Based on this average, participants were stratified as follows: anatomy high-performers (n=32; mean=88.68%) and anatomy low-performers (n=20; mean=75.83%). The non-participant subcategories were as follows: anatomy high-performers (n=21; mean=88.17%) and anatomy low-performers (n=31; mean=75.70%). 

The mean CV physiology final exam class average was 85.82%. The mean CV exam score of the 54 participants was significantly higher than the mean of the 52 non-participant CV exam scores (87.56% vs. 83.01%; p < 0.001). Both low- and high-performing TBL-US participants had statistically significantly higher average scores than their non-participant counterparts using the student t-test. There was a mean difference of +3.27% (p<0.038) on the CV exam among the high-performing students (participants n=31; u=89.22% vs. non-participants n=21; u=85.95%) and a +4.9% difference (p<0.039) among the low-performing students (participants n=20; u=85.92% vs. non-participants n=31; u=81.02%). There was no significant difference in mean structure and function exam scores among the 52 randomly selected non-participants in either the high-performer subcategory (p=0.632) or the low-performer subcategory (p=0.936).

When considering the potential bias of previous Structure and Function exam performance, multiple regression analysis in both the adjusted and unadjusted models yielded TBL participation still had a moderately statistically significant impact on the final CV exam scores (R=0.556 vs. R=0.339, respectively). Further stratification into high- and low-performance groups was not statistically significant since the Structure and Function exam scores were also included in the model.

**Table 1:** Mean Difference in Survey Response Scores Among All Participants (N=54)

**Topic**	**Median Pre-Survey Score [IQR]**	**Median Post-Survey Score [IQR]**	**Median Difference**	**Test Statistic (Z)**	**p-value**	**r**	**Effect size (based on** **r** **)**
Confidence in understanding of cardiovascular physiology from introductory physiology course	3 2, 3	4 4, 5	1	-5.406	< 0.001	-0.520	Large
Importance of hands-on ultrasound in learning cardiovascular physiology	4 3, 4	5 4, 5	1	-5.349	< 0.001	-0.514	Large
Excitement for learning more hands-on POCUS skills in the future	3 [2.75, 4]	4 4, 5	1	-4.663	< 0.001	-0.449	Moderate
Confidence in understanding of cardiovascular anatomy from introductory anatomy course	3 3, 4	4 4, 5	1	-4.641	< 0.001	-0.447	Moderate
Confidence in team-working skills after participating in TBL-US	3 3, 4	4 4, 5	1	-4.628	< 0.001	-0.445	Moderate
Importance of group activity in learning cardiovascular physiology	4 4, 5	5 5, 5	1	-4.609	< 0.001	-0.444	Moderate
Confidence in communication skills after participating in TBL-US	4 3, 4	4 4, 5	0	-3.972	< 0.001	-0.382	Moderate
Perceived importance to learn hand-on POCUS skills in medical school	3 3, 4	4 [3.75, 5]	1	-3.957	< 0.001	-0.381	Moderate
Importance of group activity in learning hands-on POCUS skills	4 3, 4	5 4, 5	1	-3.294	< 0.001	-0.317	Moderate
Importance of group activity in learning cardiovascular anatomy	4 3, 4	4 4, 5	0	-3.083	0.002	-0.297	Small
Importance of hands-on POCUS in learning cardiovascular anatomy	4 3, 5	5 4, 5	1	-2.585	0.01*	-0.249	Small

^a^Rated on a 5-point Likert Scale (1 = Strongly Disagree, 5 = Strongly Agree)

*This test was not statistically significant following the Bonferroni correction

### Survey Results

The Wilcoxon Signed-Rank nonparametric test was used to compare the pre-and post-survey results of the samples. The Bonferroni correction [0.05/11] yielded a p-value of 0.005 for each test. Additionally, 10 out of 11 of the results were statistically significant indicating TBL had an impact on the perception of all topics except for the importance of hands-on POCUS in learning CV anatomy. The two topics that yielded the largest effect size from the TBL workshop were an increase in confidence in understanding CV physiology initially presented from the introductory course and the increased importance of hands-on POCUS in learning CV physiology (Table 1). 

Over 90% of students indicated positive team dynamics regarding communication, problem-solving, and critical thinking skills during team discussions. After the session, 98% of students either “strongly agreed” or “somewhat agreed” that TBL is an effective tool for teaching POCUS and that TBL-US should be implemented into the curriculum. As well, 86% of students felt that it was either “extremely important" or “very important” for medical students to learn POCUS compared to 72.6% of students before the session (p < 0.05). 96.2% of students indicated that they were “comfortable” or “extremely comfortable” having a near-peer teach them, and 98% indicated they found the TBL component to be valuable. 

In the free-response section, the three most cited strengths of the TBL-US were “teaching by peer facilitators” (n=14), “additional practice questions” (n=13), and “hands-on POCUS application” (n=11), while the three most frequent criticisms were “discrepancy between session length” (n=5), “could not correlate physiology and US” (n=4), and “wanting more resources” (n=3).

## Discussion

The objective of the study was to investigate if a session on cardiac POCUS skills delivered to first-year medical students could enhance comprehension of CV anatomy and physiology while encouraging group collaboration. Few interventions have used TBL to teach POCUS. The literature indicates that TBL can be superior to conventional education methodologies in teaching musculoskeletal POCUS skills and non-inferior in teaching cardiac echocardiography skills to undergraduate medical students 27, 28. POCUS is a method particularly conducive to a TBL approach because it is a practical skill that requires feedback from adept operators. It requires both technical acumen and cognitive interpretation, making TBL an effective vehicle for teaching it.

Benefits of Near-Peers

Access to faculty and residents who are well-trained in cardiac POCUS can be difficult due to factors such as scheduling and availability of expertise. Literature has shown that trained medical students can effectively teach clinical skills and create a more comfortable learning environment for their underclass peers to ask questions and engage with the material while decreasing faculty supervision burden 17, 25, 29, 30, 31, 32, 33. Similar results were echoed in this study. Training medical students to teach clinical skills can create a self-sustaining pool of peer-facilitators at any institution 29, 30, 31, 32. While not addressed in this study, future studies could examine the potential increase in knowledge consolidation of near-peer facilitators.

Knowledge Assessments 

Where the IRAT assessed the students’ initial knowledge acquisition of POCUS and physiology concepts from advanced preparation resources, the post-session assessment gauged retention and application. The idea of collaborative testing as a component of TBL is a well-known pedagogical strategy [373]. Participants scored significantly higher on the post-session knowledge assessment than the pre-session knowledge assessment across all three concepts, indicating short-term learning and retention. 

TBL-US participants in both the “high-performer” and “low-performer” sub-groups scored higher on the CV final exam compared to non-participants. In addition, the significant improvement of low-performing participants compared to non-participants suggests that TBL-US may also enhance learning and application of foundational concepts. Overall, these results are comparable to other studies involving preclinical POCUS education, showing that TBL-US is not only effective, but also adds synergistic benefits when combined with POCUS imaging compared to TBL alone 1, 5, 7, 10, 13, 18, 19, 21, 22, 23, 24, 34 .

Student Surveys

Most students found that TBL-US enhanced their understanding of CV anatomy and physiology. Students felt more confident in their understanding of anatomy and physiology, basic POCUS skills, and communication and teamwork abilities after participating. Many students also perceived group learning to be effective for consolidating knowledge of the material. Almost all students noted that multiple viewpoints were both elicited and respected. 

## Limitations

TBL-US participation was a voluntary activity, which contributed to selection bias and the reduced number of participants compared to the eligible pool. Participants might have been more self-motivated to learn POCUS and were more enthusiastic about participating in the session. Furthermore, students who were interested but perceived a lack of availability may not have participated. As a result, the attitudes and perceptions collected may not reflect those of the entire class. While TBL-US materials may have been shared more widely amongst the students, given the temporal proximity to the exam, materials directly provided by the course director would have been preferentially used. 

Additionally, the optimal facilitator-to-student ratio would have been 1:5 to promote interactivity, accessibility to ultrasound machines, and direct participation time 5, 12. However, with only four machines available, the facilitator-to-student ratio was 1:7. Technological advances such as mirroring methods that allow screen sharing and video streaming directly from the ultrasound machine to computers and mobile devices may be one avenue to mitigate this. Lastly, as this session was evaluated at a single institution, results may not be generalizable to all institutions at all geographic locations.

## Conclusion

With an increased demand for POCUS education, we piloted a novel POCUS educational module within the preclinical undergraduate medical curriculum. Our session was associated with (1) increased academic performance in both the TBL post-assessments and the preclinical CV physiology exam, (2) improved self-reported confidence and comfort level in POCUS, and (3) a continued interest in pursuing further POCUS education. After this study, a POCUS simulation program was formalized and adopted to supplement the CV course. This is promising for further studying and implementing TBL and near-peers for POCUS education. Future interventions of TBL in US training augment both CV as well as other anatomy courses by leveraging FOAMed and near-peer students even in institutions with limited POCUS-trained instructors.

## Disclosure

Ultrasound machines were provided by General Electric (GE) through the GE Healthcare Grants and Sponsorships – Ultrasound Initiatives Program. The authors declare that they have no competing interests. 

## Statement of Ethics Approval/Consent

This intervention was approved by the Institutional Review Board of the Rutgers Biomedical and Health Sciences (Pro2019002087). All participants signed informed consent forms before their participation.
